# Removal of Carbapenem-Resistant Enterobacteriaceae (CRE) from Blood by Heparin-Functional Hemoperfusion Media

**DOI:** 10.1371/journal.pone.0114242

**Published:** 2014-12-03

**Authors:** Keith McCrea, Robert Ward, Steven P. LaRosa

**Affiliations:** 1 ExThera Medical Corporation, 813 Heinz Avenue, Berkeley, CA 94710, United States of America; 2 Infectious Disease, Beverly Hospital/Lahey Health System, 85 Herrick Street, Beverly, MA 01915, United States of America; University of Pittsburgh, United States of America

## Abstract

Bloodstream infections due to Carbapenem-Resistant Enterobacteriaceae (CRE) are becoming more frequent and are associated with a high mortality. At present, combination antimicrobial therapy yields the best outcomes, but treatment options are limited. Many bacteria utilize heparan sulfate to bind to human cells. We studied the ability of a biomimetic device composed of polyethylene beads with endpoint-attached heparin to bind both sensitive and (CRE) E. coli and *Klebsiella pneumoniae* from spiked blood samples. Greater than 90% of susceptible, *E. coli*, CRE *E. coli* and CRE *Klebsiella* were removed by the beads. Future studies in human bacteremia with this technology are planned.

## Introduction

Enterobacteriaceae are Gram negative bacteria that are normal inhabitants of the intestinal microbiota. These organisms are responsible for catheter-related bacteremias, nosocomial pneumonias, and urinary tract infections. Members of this family that cause infection include *E. coli* and *Klebsiella pneumoniae*. Increasing antimicrobial resistance has been observed in Enterobacteriaceae. Carbapenem resistant Enterobacteriaceae (CRE) have been reported in 33 U.S states and 9 countries [1]. Data from the National Healthcare Safety Network (NHSN) indicate that the percentage of (CRE) *Enterobacteriaceae* has increased from 1.2% in 2001 to 4.2% in 2011. Bacteremia occurs in approximately 10% of CRE infections with an associated mortality of 50% [2].

The treatment options for bacteremia due to CRE organisms are limited. Treatment options include agents with poor blood levels, Tigecycline, or those with significant associated toxicity, Colistin or aminoglycosides. A potential adjunctive therapy for CRE-associated bacteremia would be pathogen removal via an extracorporeal cartridge. Our approach is to develop a biomimetic device that capitalizes on the known attachment of many species of bacteria to heparan sulfate on cells. Heparin is structurally similar to heparan sulfate and binds many of the same adsorbates. We have developed covalently end-point attached heparin-coated ultrahigh molecular weight polyethylene (UHMWPE) as an adsorption media. The following study examines the ability of this technology to remove both sensitive and cabapenemase-resistant members of the *Enterobacteriaceae* family.

## Materials and Methods

### Covalent End-Point Attachment of Heparin and Test Articles

Ultrahigh molecular weight polyethylene (UHMWPE) beads, with an average diameter of 0.3 mm were supplied by DSM Biomedical (Berkeley, USA). Pharmaceutical grade heparin and polyethyleneimine (PEI) are purchased from Scientific Protein Laboratories (Waunakee, Wisconsin, USA) and BASF (Ludwigshafen, Germany) respectively. All chemicals used are of analytical grade or better.

Immobilization of heparin onto the beads was performed as described by Larm et al [3]. Briefly, the UHMWPE surface was heparinized using the general procedure described below. The surface was etched with potassium permanganate in sulfuric acid to hydrophilize the beads. Reactive amino functions are introduced by treatment with polyethylenimine (PEI). End-point attachment (EPA) to the aminated surface of native heparin is done by reductive amination, utilizing the aldehyde function in the reducing terminal residue of partially, nitrous degradation of native heparin. The resulting PE-beads, with covalently end-point attached heparin, were then sterilized with ethylene oxide (ETO).

The sterilized beads (0.6 g) were then packaged in 2.5 mL test filter syringes (Mobicol, Germany) with 100 micron top and bottom porous retaining plates. A total of three filter syringes were prepared for each pathogen.

### Surface Heparin Loading and Activity

The surface loading heparin was determined using the MBTH method [3]. To verify that the covalent end-point attached heparin maintained its ATIII activity, the surface was characterized using Kinetichrome™ Heparin Anti-Xa Activity Kits (Provision Kinetics, Arlington WI). The general procedure for the Anti-Xa test is as follows. A measured quantity of heparin beads are first suspended in PBS buffer at 37C. ATIII in buffer is then pipetted into the suspension and allowed to form a surface bound ATIII/Heparin complex. An excess of Anti-Xa is then added to the suspension and only a portion binds to the surface ATIII/Heparin complex. Finally, a chromogenic Anti-Xa substrate (which is a peptide with affinity to anti-Xa) is then added to the solution to react with the residual Anti-Xa in solution. The concentration of the Anti-Xa/Substrate is then determined using a UV/Vis spectrometer and the heparin surface activity is calculated.

### Microbiology

The microbiology testing was performed at Antimicrobial Test Laboratories (ATL). For this study, the removal of high concentrations of *Enterobacteriaceae* suspended in defibrinated horse blood was tested. The bacteria tested included drug susceptible *E. coli* ATCC 8739 *and* carbapenem-resistant *E. coli ATCC* BAA-2469, drug susceptible *K. pneumoniae* ATCC 13883 and carbapenem resistant *K. pneumoniae ATCC* BAA-2146. The bacteria were cultured using standard methods and diluted in defibrinated horse blood. The targeted CFU/mL concentration was typical for antimicrobial testing and ranged between 10^5^ and 10^6^ CFU/mL. The provided filter syringes were primed with saline. An aliquot of blood with a bacterial concentration of ∼10^6^ CFU/mL was then passed through the filter syringe by gravity flow, collected, and analyzed. The filtrate was neutralized and enumerated and reduction in bacterial numbers determined (CFU/ml) based on an initial enumeration of the inoculums.

## Results

### Loading and Activity

MBTH analysis indicated a heparin loading of 2.0 mg per gram of beads. Activity analysis by Anti-XA assay determined a heparin activity of 0.3 U/mg of beads. No leaching of heparin from the surface was detected, confirming that the heparin is covalently attached.

### Removal of Enterobacteriaceae from Blood by Surface Heparin

Both drug susceptible and carbapenem resistant bacteria were suspended in defibrinated blood and passed through test filters containing 0.6 g of heparinized UHMWPE beads. The data from this study is summarized in Table 1. A total of two ml of blood were used for each bacteria with starting concentrations (CFU/ml) ranging between 1.40×10^5^ (CRE *K. pneumoniae*) to 6.15×10^5^ (*E. coli*). After passing the blood over the heparinized beads, the remaining concentrations of bacteria in blood were enumerated. The percent reduction for *E. coli*, *K. pneumoniae*, CRE *E. coli*, and *CRE K. pneumoniae* were 99.75%, 36.43%, 99.93%, and 99.94%, respectively.

**Table 1:** Removal of sensitive and CRE bacteria by heparinized beads.

Test Microorganism	Sample	Replicate	Replicate CFU/ml	Average CFU/ml	Percent Reduction compared to Time Zero	Log Reduction Compared to Time Zero
*E. coli ATCC 8739*	Time Zero	1	6.05E+05	6.15E+05	N/A	
		2	5.80E+05			
		3	6.60E+05			
	Heparinized Media	1	1.62E+03	1.54E+03	99.75%	2.6
		2	1.63E+03			
		3	1.37E+03			
*K. pneumoniae ATCC 13883*	Time Zero	1	4.15E+05	4.02E+05	N/A	
		2	3.70E+05			
		3	4.20E+05			
	Heparinized Media	1	2.96E+05	2.55E+05	36.43%	0.2
		2	2.56E+05			
		3	2.14E+05			
*E. coli ATCC BAA-2469 (CRE)*	Time Zero	1	2.65E+05	2.57E+05	N/A	
		2	2.30E+05			
		3	2.75E+05			
	Heparinized Media	1	1.55E+02	1.90E+02	99.93%	3.13
		2	2.15E+02			
		3	2.00E+02			
*K. pneumoniae ATCC BAA-2146 (CRE)*	Time Zero	1	1.30E+05	1.40E+05	N/A	
		2	1.45E+05			
		3	1.45E+05			
	Heparinized Media	1	4.50E+01	7.83E+01	99.94%	3.25
		2	1.05E+02			
		3	8.50E+01			

To better understand the capacity of the heparinized media, the total adsorbed bacteria per gram of heparinized beads were calculated for each test and summarized in Table 2. The data indicates that a single gram of heparinized beads with an average size of 300 microns adsorbed 2.04×10^6^ CFUs of *E. coli*, 4.88×10^5^ CFUs of *K. pneumoniae*, 8.56×10^5^ CFUs of CRE *E. coli*, and 4.66×10^5^ CFUs of CRE *K. pneumoniae*.

**Table 2:** Total adsorbed bacteria per gram of heparinized beads.

	Test Medium	Starting Concentration (CFU/ml)	% Removed by 0.6 grams of media	Adsorbed Bacteria (CFU/g media)
*E. coli ATCC 8739*	2 ml Defibrinated Blood	6.15E+05	99.75	2.04E+06
*K. pneumoniae ATCC 13883*		4.02E+05	36.43	4.88E+05
*E. coli ATCC BAA-2469 (CRE)*		2.57E+05	99.93	8.56E+05
*K. pneumoniae ATCC BAA-2146 (CRE)*		1.40E+05	99.94	4.66E+05

## Discussion

This study shows that a large concentration of carbapenem-resistant Enterobacteriaceae (CRE) suspended in blood can be removed by heparin-functional adsorption media. Extrapolating from these *in-vitro* results, a single gram of media can bind over 460,000 CRE *K. pneumoniae* CFUs and over 850,000 CRE *E. coli* CFUs. To put this data into perspective, a high bacterial load in a bacteremia patient may be 100 CFU/mL. If this is scaled up to the blood volume of an average adult (5 Liters), there would be a *total* of 500,000 CFUs circulating in the blood of a bacteremia patient. For this reason, it is not difficult to engineer a broad spectrum sorption hemoperfusion device of reasonable size (based on our heparin-functional media) to provide an adjunctive treatment for CRE bacteremia.

We studied both CRE and susceptible *E. coli* and *K. pneumoniae*. While the percent reduction of bacteria was lower for susceptible *K.pneumoniae* than CRE Klebsiella (Table 1), the affinity of the bacteria to beads as measured by adsorbed CFU/g of media, was similar (Table 2). The affinity of susceptible *E. coli* for the beads was greater than twofold that of the CRE *E. coli*. It is possible that the difference may be due to adhesin expression. Type 1 fimbriae and OmpA have been implicated in the attachment to endothelial cells. A recent publication indicates that the binding mechanism is likely the same for both drug susceptible and drug resistant Enterobacteriaceae [4]. However, the ratio of the expressed fimbrae may be different and could for different affinity towards our heparin media.

The data from this study builds upon what has been observed with Staphylococci with this technology. Polyethylene beads coated with end-point attached heparin removed> 65% of *Staphylococcus aureus* including MRSA from inoculated whole blood through a miniature column after a single pass [5]. Bacteria eluted from the beads were viable indicating that potentially pro-inflammatory cellular components of (dead) bacteria, are not released into the blood. [5]. These viable bound bacteria could transmit antibiotic resistance factors yet the newly resistant bacteria would likely be removed by the column. It has been reported in the literature that over 50 different pathogens target heparan sulfate (HS) as an initial attachment site during their pathogenesis [6]. The potential exists for the creation of a broad spectrum pathogen removal system in sepsis prior to final identification of the pathogen.

A potential adjunctive therapy specifically for CRE bacteremia that involves pathogen removal may help overcome many of the limitations in treating bacteremia caused by these organisms. Combination therapy for these infections is associated with improved outcomes yet often these organisms are sensitive to only 1 agent [7]. In one study from Israel 75% of patients with CRE-*Klebsiella pneumoniae* bacteremia received inappropriate initial antimicrobial therapy [8]. In a study of CRE- Klebsiella bloodstream infections in the US, the median number of days to a negative blood culture was 4 with a range of 1–18 days [9].

This study has limitations. This was an *in vitro* study using defibrinated horse blood making it impossible to extrapolate to a benefit in patients. The concentration of bacteria spiked into the blood was higher than that seen in typical human bacteremia making it unclear how this technology would fair with lower bacterial inoculum. Lastly, the experiments were done in the absence of concomitant antibiotic therapy. Only a clinical trial of hemoperfusion over a filter with this technology in patients with CRE bacteremia will answer these questions.
